# Attenuation of *Pseudomonas aeruginosa* infection by INP0341, a salicylidene acylhydrazide, in a murine model of keratitis

**DOI:** 10.1080/21505594.2020.1776979

**Published:** 2020-06-07

**Authors:** Prerana Sharma, Mikael Elofsson, Sanhita Roy

**Affiliations:** a Prof. Brien Holden Eye Research Centre, LV Prasad Eye Institute, Hyderabad, India; b Department of Animal Biology, University of Hyderabad, Hyderabad, India; c Department of Chemistry, Umeå University, Umeå, Sweden

**Keywords:** Antimicrobial peptides, type III secretion system, *Pseudomonas aeruginosa*, reactive oxygen species, corneal epithelial cells, INP0341

## Abstract

Pseudomonas aeruginosa

is an opportunistic pathogen and a major cause of corneal infections worldwide. The bacterium secretes several toxins through its type III secretion system (T3SS) to subvert host immune responses. In addition, it is armed with intrinsic as well as acquired antibiotic resistance mechanisms that make treatment a significant challenge and new therapeutic interventions are needed. Type III secretion inhibitors have been studied as an alternative or in accompaniment to traditional antibiotics to inhibit virulence of bacteria. In this study, INP0341, a T3SS inhibitor, inhibited cytotoxicity by *P. aeruginosa* toward human corneal epithelial cells (HCEC) at 100 μM without affecting bacterial growth in the liquid media. An increased expression of antimicrobial peptides and reactive oxygen species generation was also observed in cells exposed to *P. aeruginosa* in the presence of INP0341. Furthermore, INP0341 efficiently attenuated corneal infection by *P. aeruginosa* in an experimental model of murine keratitis as evident from corneal opacity, clinical score and bacterial load. Thus, INP0341 appears to be a promising candidate to treat corneal infection caused by *P. aeruginosa* and can be further considered as an alternative therapeutic intervention.

## Introduction

*Pseudomonas aeruginosa* is a gram-negative bacterium, ubiquitous in nature and a major opportunistic human pathogen. Corneal infections caused by *P. aeruginosa* are associated with both trauma and contact lens use and are a foremost cause of blindness worldwide [1]. In the cornea, *P. aeruginosa* activates the Toll like receptors (TLRs) that results in prompt production of cytokines and chemokines, recruitment of immune cells to the cornea and development of corneal opacity [2]. The corneal epithelium provides the first line of defense against invading bacteria [3] and the host immune response to *P. aeruginosa* is regulated by TLR4-MD-2 and TLR5 leading to an elevated expression of proinflammatory cytokines and antimicrobial peptides (AMPs) [2,4–6]. One of the fundamental virulence factors of *P. aeruginosa* is the type III secretion system (T3SS) which consists of a syringe-like apparatus that functions in a highly controlled manner to transport bacterial toxins and other proteins into the host cells [7] and amend different functions of the host to survive [8]. We and others have recently shown that wild-type PAO1 subverts the host immune responses including AMP expression [6] and attenuates generation of reactive oxygen species (ROS) in neutrophils and epithelial cells by its T3SS [6,9].

*P. aeruginosa* infections are increasingly concerning with their rise in antibiotic resistance. In contrast to other gram-negative bacteria, *P. aeruginosa* is less vulnerable to various antibiotics due to low penetrance across their outer membrane and the presence of several multi-drug efflux pumps and intrinsic β-lactamases [10,11]. To make the situation worse, *P. aeruginosa* can form biofilms that have reduced susceptibility to antibiotics [12]. Thus, it becomes important to identify and study novel therapeutic agents that are effective against *P. aeruginosa*.

T3SSs are highly conserved in several gram-negative pathogenic bacteria and play important roles by secreting toxins into the host cells and thus contributes to pathogenesis [7]. Therefore, they are considered important targets for development of new anti-infective agents. T3SS inhibitors are expected to abolish bacterial virulence without directly killing the bacteria and thus reduce selective pressure that leads to the development of resistance. To date, several small compounds have been developed as T3SS inhibitors and salicylidene acylhydrazides are extensively studied among them. INP0010 [13], INP0400 [14] and INP0007 [15] are examples of salicylidene acylhydrazides that effectively inhibit the T3SSs of several pathogens including *Salmonella, Shigella* and *Yersinia*. For this study we have selected INP0341 as it was earlier shown to be effective against *P. aeruginosa* [16]. It is also known to attenuate the infectivity of *C. trachomatis* both *in vitro* and *in vivo* [17,18]. Uusitalo *et al*. have reported INP0341 to be effective in prevention of biofilms formed by *Pseudomonas* and to attenuate *P. aeruginosa* infection in a burn wound model in Balb/c mice[16].

Herein we demonstrate that INP0341 prevents cytotoxicity induced by *P. aeruginosa* in human corneal epithelial cells and causes increased expression of antimicrobial peptides and reactive oxygen species generation in response to *P. aeruginosa*. In addition, topical application of INP0341 inhibits bacterial growth and facilitates bacterial clearance in a murine model of *P. aeruginosa* keratitis.

## Materials and methods

### INP0341

INP0341[19] was synthesized as described previously and analytical data were in agreement with those previously reported. Stock solutions of INP0341 (25 mM) were prepared in dimethylsulfoxide (DMSO), stored under dark and dry conditions as described[16]. An intermediate 5 mM solution was made in 50% aqueous DMSO, from which the working solutions were prepared further.

### Bacterial culture

PAO1[20], the mutant strain PAO1*ΔpscC*[21] and two clinical isolates of *P. aeruginosa* were used in this study. For identification of the clinical isolates, corneal ulcer materials were collected aseptically and investigated following the Institute protocol as described earlier[22]. Briefly, ulcer materials were placed on glass slides for Gram staining and were inoculated in different specific media for bacterial cultures. The pure homogenous culture was then subjected to Vitek 2 compact (bioMerieux, France) analysis for identification of the bacterium along with Gram staining and series of biochemical tests. All strains of *P. aeruginosa* were grown as described earlier[23]. In brief, bacteria were subcultured from overnight culture in Brain Heart Infusion broth (HiMedia Laboratories, West Chester, USA), washed twice in 1X phosphate buffered saline (PBS), centrifuged at 10,000 rpm for 10 min, and resuspended in 1X PBS. Dilutions of the sample were done with serum free media for the final inoculums.

### Culture of HCEC

Immortalized human corneal epithelial cells (HCEC) 10.014 pRSV-T[24] were maintained in keratinocyte serum free media containing bovine pituitary extract and recombinant human epidermal growth factors (Invitrogen, Carlsbad, USA) at 37°C and 5% CO_2_ and cultured as mentioned before. To study the AMP expression, HCEC were grown in 12-well plates (1 x 10^5^ cells/well) and infected with PAO1 in the presence or absence of INP0341 for 4 h after which cells were processed further.

### Toxicity of INP0341 against HCECs

Cytotoxicity of INP0341 toward HCEC was determined quantitatively by measuring the release of lactate dehydrogenase (LDH) into the culture media using CytoTox nonradioactive cytotoxicity assay kit (Promega, Madison, USA) following the manufacturer’s protocol. Briefly, cells were grown to confluency and 50 μM (1% DMSO), 100 μM (2% DMSO), 250 μM (5% DMSO) and 500 μM (10% DMSO) of INP0341was added in triplicate and incubated for 6 h. Cells incubated with Triton X-100 were used as a positive control. The culture supernatant was used for LDH estimation by a colorimetric assay, absorbance was recorded at 490 nm[25].

### Cell toxicity induced by *P. aeruginosa*

PAO1 or PAO1*ΔpscC* was grown overnight, and subcultured to an OD_600_ of 0.2 (10^8^ colony forming units/ml, cfu/ml), centrifuged and resuspended in cell culture medium. HCECs were exposed to PAO1 in the presence or absence of INP0341 (100 μM, 2% final DMSO concentration), or PAO1*ΔpscC* at a multiplicity of infection (MOI) 10:1 (bacteria:cells) for 6 h, washed and imaged using phase contrast microscope. Cell death was measured by LDH assay using CytoTox nonradioactive cytotoxicity assay kit (Promega, Madison, USA) following the manufacturer’s protocol. Cells were fixed with 4% paraformaldehyde and stained with Alexa Fluor 488 phalloidin (ThermoFisher Scientific, Waltham, USA) for 15 min and were counterstained with DAPI (Vector Laboratories, Burlingame, USA) to visualize the nucleus. Images of the cells were captured on a fluorescent microscope (Olympus IX73, Zeiss, Germany) using the 100X objective.

### Assay for reactive oxygen species

1 x 10^4^ cells per well were cultured overnight in 96-well plates and infected with PAO1 in the presence or absence of INP0341 (100 μM, 2% final DMSO concentration) for 2 h at a MOI of 10. Cells were also infected with Δ*pscC* mutant PAO1 for the same period of time. After washing with 1X PBS, cells were incubated with 2ʹ7’-dichlorodihydrofluorescein diacetate dye containing media (H_2_CFDA; Invitrogen, Carlsbad, USA) for 15 min. The cells were further washed, media was added and the cells were observed under a fluorescent microscope (Olympus IX73, Zeiss, Germany) using a 10X objective. In separate experiments, cells were exposed to bacteria as mentioned above and the fluorescence intensity of the H_2_CFDA dye was measured quantitatively by SpectraMaxM3 (Softmax Pro 6.3).

### Murine models of corneal infection

C57BL/6 mice (6–8 week old) were purchased from Cyagen Biosciences and experiments were performed at Vivo Bio Tech Ltd, Hyderabad, a Contract Research Organization. All animal experiments were approved by the Institutional Animal Ethics Committee of the test facility (VB/IAEC/09/2018/281/Mouse/C57BL/6). The animals were housed in specific pathogen free conditions in microisolator cages and were treated in accordance with the guidelines provided in the ARVO Statement for the Use of Animals in Ophthalmic and Vision research. Mice were anesthetized by intraperitoneal injection of ketamine (8.7 mg/ml) and xylazine (0.5 mg/ml) at a dose of 0.01 ml/g body weight and the corneal epithelium was abraded with three parallel 1 mm scratches using a 26 gauge needle and separated in two random groups. A 2.5 μL aliquot containing approximately 1 × 10^5^ PAO1 was added topically to one eye, and 1X PBS was added to the fellow eye of one group, and mice remained in this position for 5 min. To the second group, 5 μL of 500 μM of INP0341 in 10% DMSO was added immediately after addition of PAO1 to one eye, and only INP0341 (in 10% DMSO) was added to the other eye. 5 μL of DMSO was also added to the infected group untreated with INP0341. The second dose of INP0341 was added topically 6 h post infection to the second group. Mice were euthanized and examined under a stereomicroscope for corneal opacification, ulceration, or perforation 24 h post infection. Clinical scores for the opacity were determined in a blinded fashion according to the scale earlier reported[26]. To measure cfu, whole eyes were homogenized in sterile 1X PBS using a tissue homogenizer (Genetix Biotech, Hyderabad, India) and serial dilutions were plated on LB agar plates, and cfu was counted manually.

### Histology and immunohistochemistry

Eyes from control and infected mice were enucleated and placed in 10% formalin and corneal sections were prepared. Hematoxylin and eosin (H&E) staining was performed following deparaffinization of 5 μm corneal sections as described earlier[2].

### RNA isolation, cDNA synthesis and quantitative PCR analysis

Quantitative real-time PCR was used to determine mRNA expression of different AMP genes from murine corneas and HCEC. The primers used are shown in Table 1 . Relative quantities of mRNA expression of respective genes were normalized using the 2^−ΔΔct^ method using GAPDH as the housekeeping gene.

**Table 1.** Oligonucleotide sequences.

Gene	Sequence (5ʹ→3ʹ)
*hBD-2*	FWD:CAGCCATCAGCCATGAGG REV:TGGCTTTTTGCAGCATTTT
*hBD-3*	FWD:TCTCAGCGTGGGGTGAAGC REV:CGGCCGCCTCTGACTCTG
*S100A9*	FWD:TGGCTCCTCGGCTTTGG REV:CGACATTTTGCAAGTCATCGTC
*S100A12*	FWD:AGCATCTGGAGGGAATTGTCA REV:GCAATGGCTACCAGGGATATGAA
*LL-37*	FWD:TCGGATGCTAACCTCTACCG REV:ACAGGCTTTGGCGTGTCT
*Rnase7*	FWD:GAACACCAAGCGCAAAGC REV:CAGCAGAAGCAGCGAAGG
*GAPDH*	FWD:GATCCCTCCAAAATCAAGTG REV:GGCAGAGATGATGACCCTTTT
*mBD-1*	FWD:CCAGATGGAGCCAGGTGTTG REV:AGCTGGAGCGGAGACAGAATCC
*mBD-2*	FWD:AAGTATTGGATACGAAGCAG REV:TGGCAGAAGGAGGACAAATG
*mS100A9*	FWD:ATACTCTAGGAAGGAAGGACACC REV:TCCATGATGTCATTTATGAGGGC
*CRAMP*	FWD:GTCTTGGGAACCATGCAGTT REV:TGGTTGAAGTCATCCACAGC
*mLipocalin2*	FWD:GCAGGTGGTACGTTGTGGG REV:CTCTTGTAGCTCATAGATGGTGC
*mGAPDH*	FWD:AGGTCGGTGTGAACGGATTTG REV:GTAGACCATGTAGTTGAGGTCA

### Statistical analysis

Bar graphs represent mean and error bars represent standard error of mean (SEM). Statistical analysis was performed using either a one-way ANOVA or an unpaired *t* test (Prism; GraphPad Software). *p* values less than 0.05 were considered significant.

## Results

### INP0341 prevents cytotoxic effects of P. aeruginosa on HCECs

**Figure 1.**
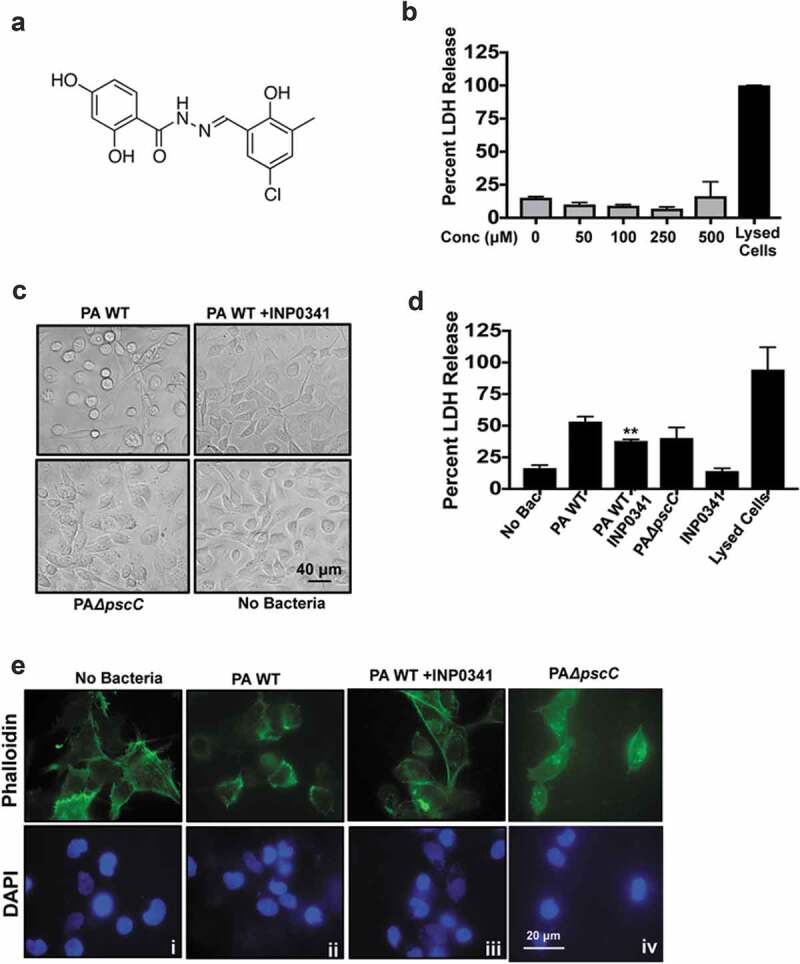
INP0341 impedes cytotoxic effects of *P. aeruginosa* on HCEC. Chemical structure of INP0341 (a). HCEC were exposed to different concentrations of INP0341 for 6 h to test its effect on cell viability using the lactate dehydrogenase (LDH) assay. Cells lysed with detergent were used as a positive control and cytotoxicity was measured as a percentage of total LDH (b). HCEC were infected with PAO1 in the presence or absence of INP0341 (100 μM), or PAO1Δ*pscC* for 6 h and cell morphology was imaged under a bright field microscope (c) and cell viability was determined by LDH assay (d). Cells were stained with phalloidin- Alexa Fluor 488 and imaged under a fluorescent microscope using a 100x objective and oil-immersion (e). The experiments were repeated at least three times.

INP0341 (Figure 1(a)) was earlier shown to reduce cytotoxicity by *P. aeruginosa* in HeLa cells[16]. Building on these results we first investigated if INP0341 exerted any cytotoxic effect on HCEC or bacteria. HCECs were incubated with increasing concentrations of INP0341 for 6 h, and cell viability was determined by measuring the release of cytosolic enzyme lactate dehydrogenase (LDH) into the culture media[27]. Cells lysed using detergent were used as a positive control. No significant cytotoxicity was observed in INP0341 treated cells even at higher concentrations (Figure 1(b)). Toxicity toward bacteria was also determined by monitoring the growth at 6 and 24 h in the presence of increased concentrations of INP0341 and no significant difference in growth was observed in the presence of higher concentrations of the inhibitor (Fig. S1). Since INP0341 was not toxic to HCECs, we then examined the effect of INP0341 on the cytotoxicity of *P. aeruginosa* toward HCECs. PAO1 is known to cause cell cytotoxicity in airway epithelial cells[28], and murine macrophages[23] . HCECs were exposed to PAO1 in the presence or absence of INP0341 (100 μM) or PAO1Δ*pscC*, and cytotoxicity was determined after 6 h by observing the cell phenotype and LDH assay. Cells exposed to PAO1 changed morphologically and about seventy percent of the cells became rounded up. This was significantly reduced in the presence of the inhibitor, and cells were of a similar phenotype to those of uninfected cells (Figure 1(c)). In comparison to the cells completely lysed by triton-X 100, PAO1 infection caused fifty percent of LDH maximum release, which was significantly lowered in the presence of INP0341 (Figure 1(d)). HCEC treated with INP0341 only showed less than 15% LDH maximum release. Several bacteria including *P. aeruginosa* cause T3SS mediated disruption of the actin cytoskeleton of the host cells[29]. To observe if INP0341 inhibits actin cytoskeleton rearrangements in cells induced by *P. aeruginosa*, HCECs were infected with *P. aeruginosa* in the presence or absence of INP0341 and stained with phalloidin-Alexa Fluor 488. We observed redistribution of the actin cytoskeleton along with cell rounding in cells infected with *P. aeruginosa* (Figure 1(e) ii). This effect was visibly inhibited in the presence of INP0341 (Figure 1(e) iii).

### Inhibition of T3SS by INP0341 enhanced AMP expression by HCEC in response to P. aeruginosa

**Figure 2.**
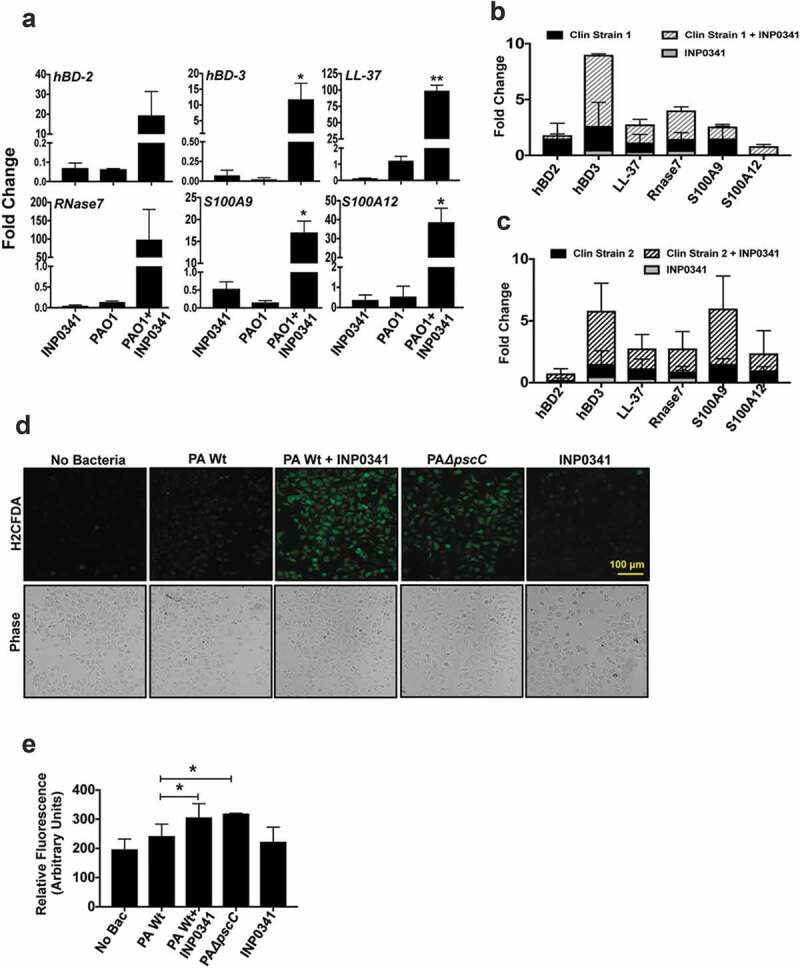
Increased expression of AMPs and generation of ROS in HCEC exposed to *P. aeruginosa* in presence of INP0341. HCEC were infected with PAO1 (a) or clinical isolates (b and c) in the presence or absence of INP0341 for 4 h, and AMP gene expression was determined by quantitative PCR using the 2^−ΔΔCt^ method. GAPDH was used as a housekeeping gene. For detection of ROS, HCEC were exposed to PAO1 in the presence or absence of INP0341 (100 μM) or PAO1Δ*pscC* for 2 h and incubated with H_2_CFDA for 15 min and then observed and imaged using a fluorescent microscope (d). The generation of ROS was also quantitated using a fluorescent plate reader (e). The experiments were done in duplicate and repeated three times. (*indicates p < 0.05).

In our earlier study we investigated the expression of AMPs from corneal scrapings of patients with *P. aeruginosa* corneal infections and saw differential expression of several AMPs, including human β defensins (hBD) 2, and 3, S100A9, S100A12 and LL-37. We also found that PAO1 subverts the expression of these AMPs in HCECs *in vitro*[6]. However, we observed that the expression of the AMPs increased significantly when cells were exposed to PAO1Δ*pscC*, a T3SS mutant that fails to transfer exotoxins to the host cells. We therefore continued to investigate if pharmacological inhibition of the T3SS has a similar effect on AMP expression. Two clinical isolates of *P. aeruginosa* along with PAO1 were included for this experiment. Both the clinical isolates were positive for *exoS, T* and *Y* genes but not *exoU*, similar to PAO1 (data not shown), and thus harbor the T3SS. HCECs were infected either with PAO1 or the clinical isolates at MOI 10 in presence or absence of 100 μM INP0341 and the expression of AMPs was determined. We found increased expression of hBD-2, hBD-3, LL-37, RNase7, S100A9 and S100A12 in HCEC in response to PAO1 in the presence of INP0341 compared to PAO1 alone (Figure 2(a)). Increased AMP expression was also observed in cells exposed to clinical isolates of *P. aeruginosa* in the presence of INP0341 compared to bacteria alone in agreement with our hypothesis (Figure 2(b,c)).

### Effect of INP0341 on PAO1 induced ROS generation by HCEC

ROS generation in response to infections is not only to kill the invading pathogen but also to mediate the host immune responses. Like several other bacteria, *P. aeruginosa* has the ability to subvert ROS generation mediated by its T3SS in neutrophils[9] and epithelial cells[6], thus reducing the phagocytic killing by cells. Therefore we investigated if INP0341 could inhibit this subversion of ROS generation in host cells. HCECs were exposed to PAO1 in the presence or absence of INP0341 for 2 h and ROS generation was determined using fluorescent probe H_2_CDFA. Similar to our earlier observations[6], we saw a minimum level of ROS in cells infected with PAO1 (Figure 2(d) ii). However, a significant increase in ROS generation was observed in HCECs infected with PAO1 in the presence of INP0341 (Figure 2(d) iii) compared to cells infected without INP0341 (Figure 2(d) ii). Increased ROS generation was also observed in PAO1Δ*pscC* infected cells (Figure 2(d) iv) compared to PAO1 infected cells as reported earlier[6]. The ROS generation was also quantitatively measured by fluorimeter, and significantly increased levels of ROS were detected in cells infected with PAO1 in the presence of INP0341 (Figure 2(e)) compared to infected cells alone.

### INP0341 attenuates P. aeruginosa infection in a murine model of keratitis

**Figure 3.**
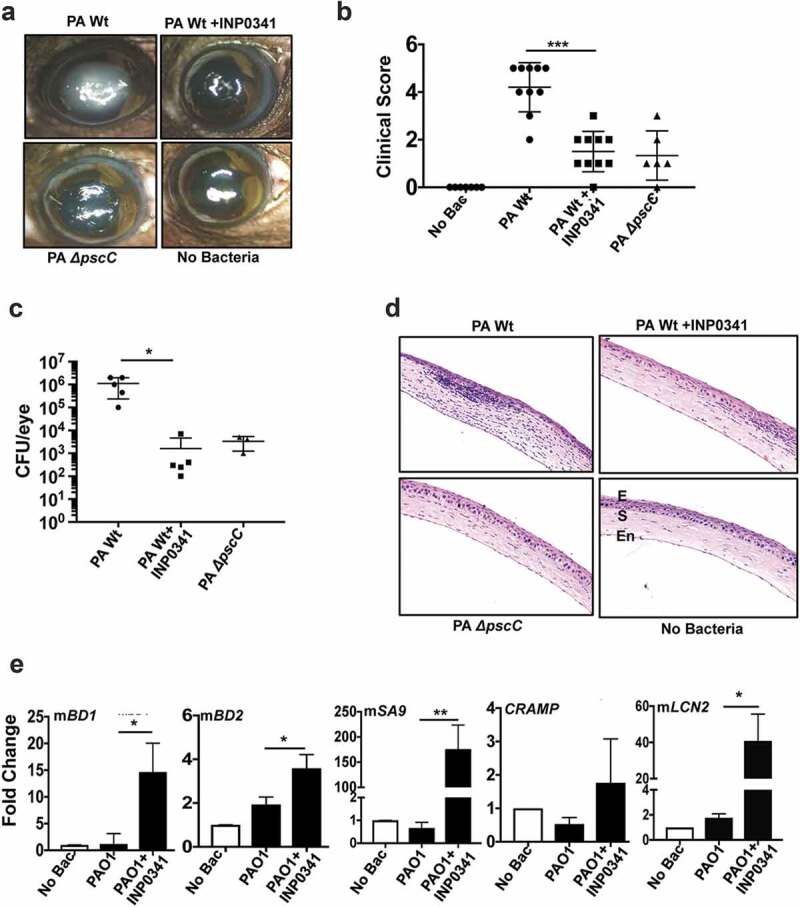
INP0341 attenuates *P. aeruginosa* infection and facilitates bacterial clearance in a murine model of keratitis. C57BL/6 mice were infected with *P. aeruginosa* and topically treated with INP0341 at 0, and 6 h post infection. Mice were euthanized 24 h post infection and representative images of corneal opacification (a) and their clinical score (b) were recorded. Cfu was measured from whole eye homogenates 24 h post infection (n = 5 mice). Data points represent individual infected corneas (c). Corneal sections were stained with hematoxylin and eosin to visualize cellular infiltration. E, epithelium; S, stroma; En, endothelium (d). Corneas (n = 3) were excised 24 h post infection and homogenized for RNA isolation and expression of AMPs was determined by quantitative PCR using 2^−ΔΔCt^method. GAPDH was used as a housekeeping gene. (*indicates p < 0.05; ** indicates p < 0.005).

To determine the effect of INP0341 in *P. aeruginosa* keratitis *in vivo*, we used our established murine model of keratitis[2] in which corneas of C57BL/6 mice were scratched and infected with PAO1. INP0341 (500 μM in 10% DMSO) was applied topically at 0, and 6 h post infection. Mice were also infected with PAO1Δ*pscC* as a separate group. Animals were euthanized after 24 h and corneas were imaged for opacification, and cfu in whole eyes were quantified after enucleation. Increased corneal opacification was detected in mice infected with wild-type PAO1, but significantly less opacity was observed in PAO1 infected mice treated with INP0341, or in mice infected with PAO1Δ*pscC* (Figure 3(a)). Significant differences were noted in the clinical scores of the corneal opacity between the groups (Figure 3(b)). Consistent with these data, cfu per eye obtained from INP0341 treated corneas were significantly lower by almost three log compare to untreated corneas that were infected with PAO1 (p < 0.0219) (Figure 3(c)). There was significantly reduced opacity and cfu in corneas infected with PAO1Δ*pscC* compared to PAO1 infected corneas as was reported earlier[30]. Hematoxylin and eosin stained sections of mice corneas showed reduced cellular infiltration in the corneal stroma of PAO1 infected mice treated with INP0341 compared to untreated infected mice (Figure 3(d)). There was also less infiltration in PAO1Δ*pscC* infected mice compared to PAO1 infected ones. Since expression of AMPs in HCECs was augmented in the presence of INP0341 in response to PAO1, we further studied the expression of AMPs in the presence of the inhibitor during corneal infections *in vivo*. Corneas were infected with PAO1 in the presence or absence of INP0341 as mentioned earlier, and AMP expression was determined 24 h post infection by QPCR. Similar to results seen with human corneal epithelial cells *in vitro*, there was reduced expression of mBD-1, mBD-2, mS100A9, lipocalin (mLCN2) and CRAMP in PAO1 infected corneas, however AMP expression increased significantly in infected corneas treated with INP0341 (Figure 3(e)).

In a subsequent experiment, infected corneas were treated either at 0 and 6 h (group I) or at 3 and 6 h (group II) post infection with INP0341 (500 μM in 10% DMSO). Mice were euthanized and corneas were imaged 24 h post infection and reduced opacity was observed in both the group I and group II mice compared to the infected group (Fig. S2A). Significant reduction in clinical scores of the opacity was observed in mice of group I compared to the infected group (p < 0.0014). Although clinical scores of mice of group II were less than the infected group, the difference was not significant (Fig. S2B). Additionally, reduced cfu were observed in mice of group I but not of group II compared to the infected group (Fig. S2C). Consistent with these data, the hematoxylin and eosin staining of corneas of group I mice show reduced cellular infiltrations in the corneal stroma compared to PAO1 infected mice (**Fig. S2D**). The infiltrations observed in the corneal sections of group II mice were more than that of group I but lower than PAO1 infected group. Taken together, these findings indicate that INP0341 is effective in controlling infection in an experimental model of *Pseudomonas* keratitis.

## Discussion

In this study, we investigated the application of INP0341, a T3SS inhibitor, for the treatment of *P. aeruginosa* keratitis in a murine model. *P. aeruginosa* employs T3SS to translocate toxins into eukaryotic cells resulting in infection. It also has a unique ability to develop antibiotic resistance[31]. The increase in antibiotic resistance has generated interests in targeting T3SS to prevent or treat infection by mechanisms distinct from those of conventional antibiotics. Furthermore, since T3SSs is typically found in pathogenic bacteria, targeting this system will not affect the vast commensal population aside[32], thus reducing the likelihood of the emergence of resistant bacteria in this population. It has been reported that hydroxyquinolines effectively inhibited T3SS in *Y. pseudotuberculosis, C. trachomatis*[33], and *P. aeruginosa* mediated lung injury[34]. Phenoxyactemaide inhibitors also selectively inhibit T3SS by presumably targeting the needle protein PscF[35]. Recently, Berube *et al*. reported that several phenoxyacetamide inhibitors could inhibit abscess formation in a murine model of *P. aeruginosa* infection[36]. Aurodox, a polyketide compound, that inhibits T3SS, was also found to be effective against *C. rodentium* induced colonic hyperplasia in a mouse model[37].

The salicylidene acylhydrazides block the T3SS in several pathogens including *Y. pseudotuberculosis* [38,39], *C. trachomatis*[40], *S. enterica*, and *P. aeruginosa*. This class of compounds has been shown to inhibit the *S. flexeneri* invasion of HeLa to induce apoptosis in macrophages *in vitro*[14], and to inhibit *S. enterica* protein secretion and invasion of Madin-Darby Canine Kidney cells[13]. Of the several salicylidene acylhydrazides, INP0341 has been extensively studied and was found to be effective against several pathogens, including *P. aeruginosa* and *C. trachomatis*. The salicylidene acylhydrazides have the capacity to chelate iron and INP0341 was shown to exhibit *in vitro* activity against *N. gonorrhoeae*[17] and HIV[41] through an iron restriction mechanism. A recent study revealed that the topical application of INP0341 significantly increased the survival of mice with burn wounds infected with *P. aeruginosa*[16]. We have extended our studies to determine the effect of INP0341 on *P. aeruginosa* in corneal epithelial cells. Here we showed that INP0341 protected corneal epithelial cells from *P. aeruginosa* infection. It has previously been shown that PAO1 inhibited host immune responses both *in vivo* and *in vitro*. Increased bacterial load was observed in the corneas of C57BL/6 mice infected with PAO1 compared with those infected with a T3SS deficient strain[30]. PAO1 was also found to impede the expression of AMPs, reduce the generation of ROS, and inhibit T3SS mediated p38 and ERK signaling in HCEC [6]. However, in the present study, a significant increase in the expression of AMPs was observed when HCECs were infected with PAO1 in the presence of INP0341. Treatment with INP0341 was also found to be effective against clinical isolates of PAO1 expressing ExoS. Furthermore, in the presence of INP0341, increased ROS generation was observed in PAO1 infected HCEC. Finally, we also demonstrated that INP0341 exerted a therapeutic effect following topical application to mouse corneas infected with PAO1. Significantly reduced bacterial load and increased expression of AMPs were observed in murine corneas infected with PAO1 in the presence of INP0341 applied at the time of infection. Although administered prophylactically and in higher doses than those required *in vitro*, our data suggested that INP0341 might be used to treat *Pseudomonas* infections as it improved bacterial clearance in infected corneas. However, further research is required to develop an optimal formulation and to determine the pharmacokinetic profile of INP0341 for ocular administration of the compound that can be useful to treat these blinding corneal infections. In conclusion, this study determines the potential of INP0341 to treat corneal infections caused by *Pseudomonas* and suggests that virulence inhibitors can be utilized for therapeutic intervention to combat antibiotic resistance.

## Supplementary Material

Supplemental Material
