# Supplementary material for: Attenuation of Pseudomonas aeruginosa infection by INP0341, a salicylidene acylhydrazide, in a murine model of keratitis
Source: Virulence. 2020 Jun 7;11(1):795–804. doi: 10.1080/21505594.2020.1776979 (PMC7567437; doi:10.1080/21505594.2020.1776979)
Supplement: Supplemental Material [file KVIR_A_1776979_SM4911.zip › Supplementary_Information.docx]

**Attenuation of *Pseudomonas aeruginosa* infection by INP0341, a salicylidene acylhydrazide, in a murine model of keratitis**

**Prerana Sharma^1,3^, Mikael Elofsson^2^, Sanhita Roy^1*^**

1. *Prof. Brien Holden Eye Research Center, LV Prasad Eye Institute, Hyderabad, India*
2. *Department of Chemistry, Umeå University, Umeå, Sweden*
3. *Department of Animal Sciences, University of Hyderabad, Hyderabad, India*

***Corresponding Author**

**Sanhita Roy, Ph.D.**

Prof. Brien Holden Eye Research Center,

L.V. Prasad Eye Institute

Hyderabad-500034, India

Email: sanhita@lvpei.org

Telephone: +91-40-30612529; Fax: +91-40-30612535

**Running Title:** INP0341 inhibits *P. aeruginosa* keratitis

**Supplementary Method**

*Determination of bacterial viability in presence of INP0341*

PAO1 was incubated with different concentration (0, 50, 100 and 250 μM) of INP0341 for 6 or 24 h in Luria-Bertani Media (MP Biomedicals, Mumbai, India) at 37°C. The bacterial growth was monitored by recording the absorbance at 600 nm by SpectraMaxM3 (Softmax Pro 6.3). The absorbance of bacterial growth without INP0341 was considered as hundred percent.

**Supplementary Figures**


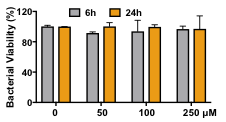


**Figure S1. Bacterial viability in presence of INP0341.** PAO1 was incubated with different concentration of INP0341 for 6 or 24 h and bacterial viability was monitored by recording the optical density at 600 nm. The absorbance of bacteria without the inhibitor was considered as 100 percent.


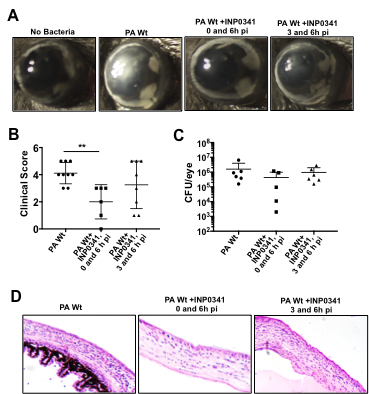


**Figure S2. Activity of INP0341 in a murine model of *P. aeruginosa* keratitis.** C57BL/6 mice were infected with PAO1 (PAWt) and topically treated with INP0341 at 0, and 6 h post infection (group I) or at 3, and 6 h post infection (group II). Mice were euthanized 24 h post infection and representative images of corneal opacification (A) and clinical scores (B) were recorded. Cfu was determined from whole eye homogenates 24 h post infection (n=6 mice). Data points represent individual infected cornea (C). Corneal sections were stained with hematoxylin and eosin to visualize cellular infiltration (D). (*indicates p<0.05; ** indicates p<0.005)
