# Supplementary figures and images for: Attenuation of Pseudomonas aeruginosa infection by INP0341, a salicylidene acylhydrazide, in a murine model of keratitis
Source: Virulence. 2020 Jun 7;11(1):795–804. doi: 10.1080/21505594.2020.1776979 (PMC7567437; doi:10.1080/21505594.2020.1776979)

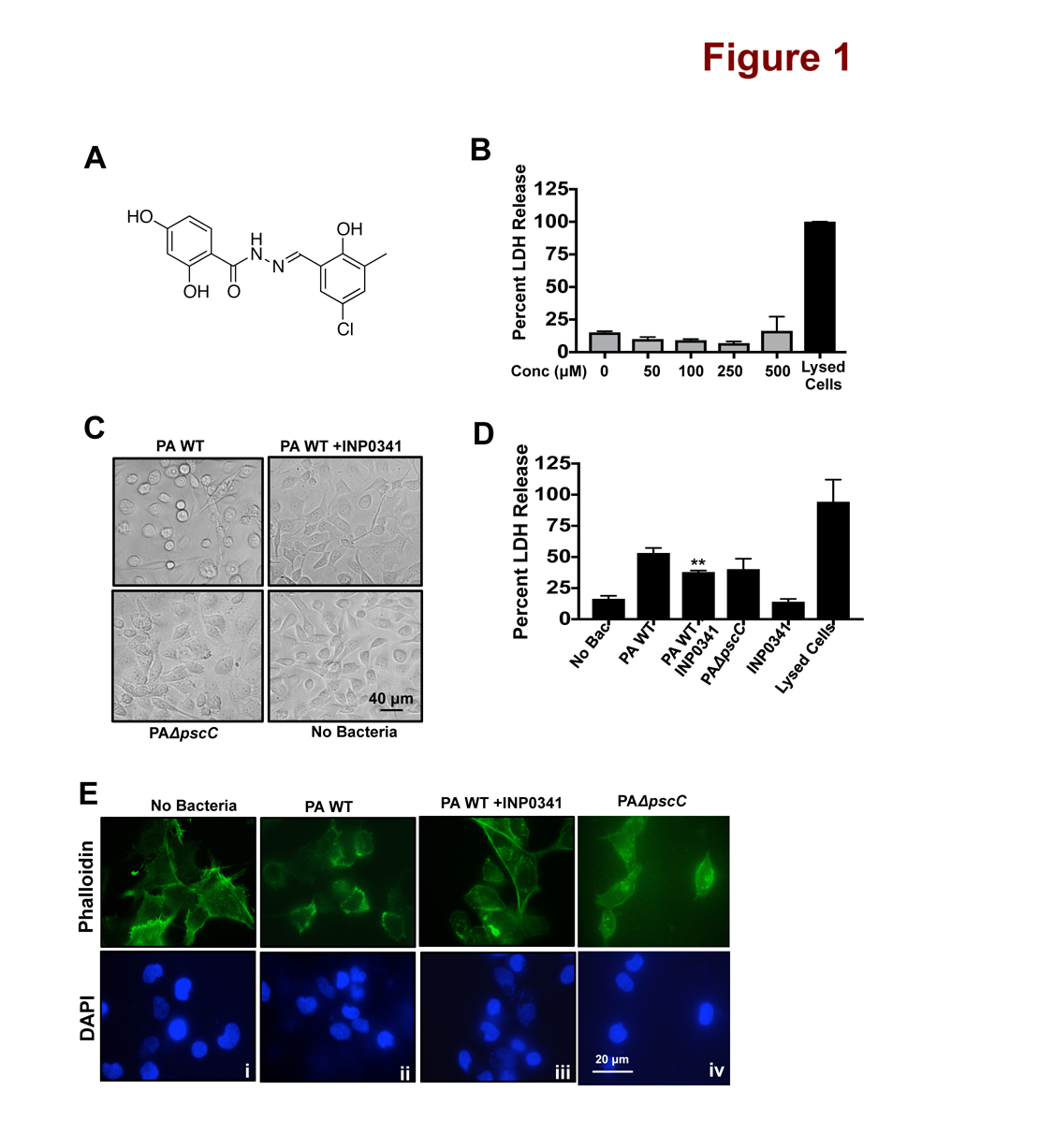

Supplement: Supplemental Material [file KVIR_A_1776979_SM4911.zip › Supplementary_Figure_1.tif]

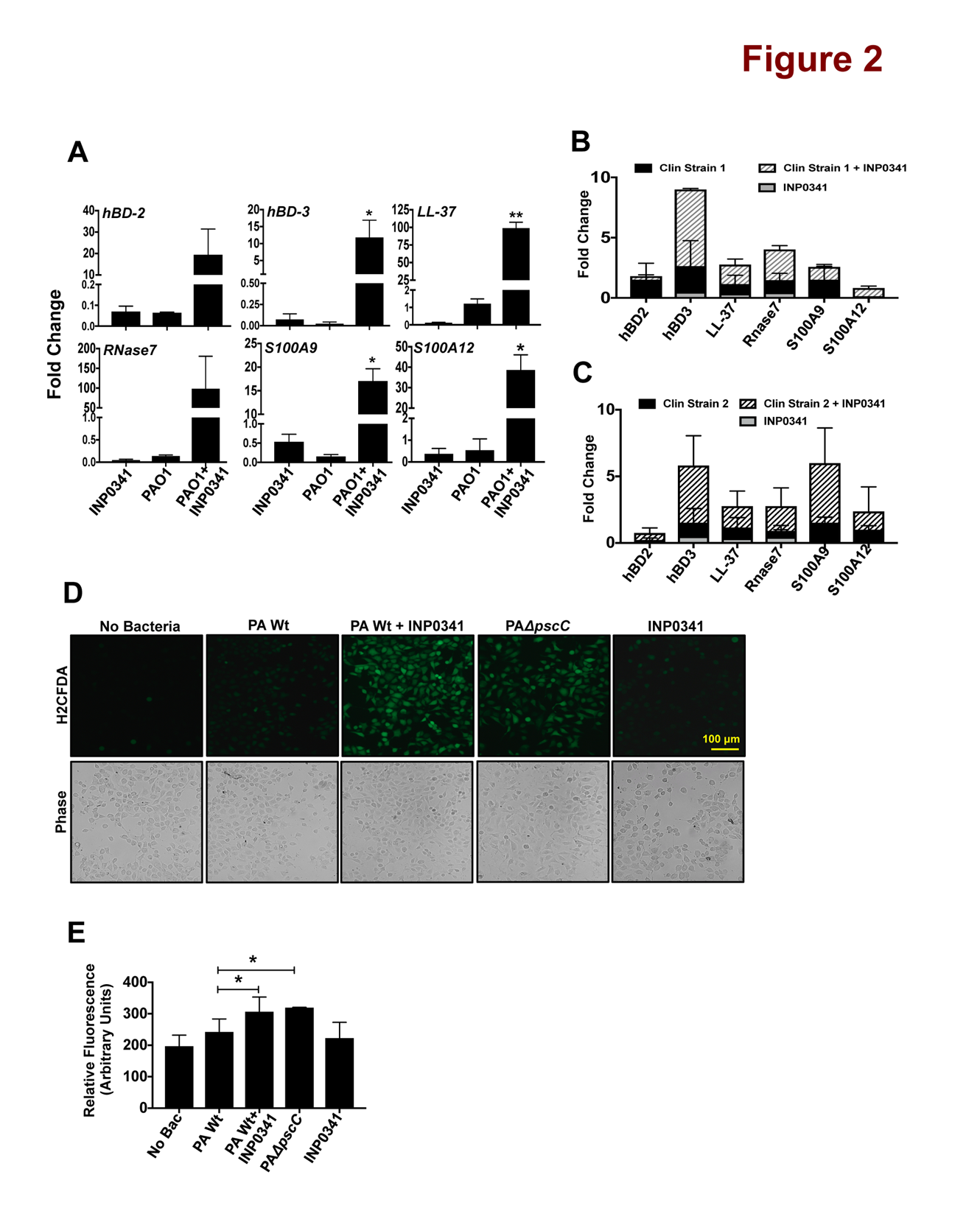

Supplement: Supplemental Material [file KVIR_A_1776979_SM4911.zip › Supplementary_Figure_2.tif]
